# A comprehensive approach to rehabilitation interventions following breast cancer treatment - a systematic review of systematic reviews

**DOI:** 10.1186/s12885-019-5648-7

**Published:** 2019-05-20

**Authors:** U. Olsson Möller, I. Beck, L. Rydén, M. Malmström

**Affiliations:** 1 Department of Nursing and Integrated Health Sciences, Faculty of Medicine, Kristianstad University, Kristianstad, Sweden; 2 The Institute for Palliative Care, Lund University and Region Skåne, Lund, Sweden; 3 Department of Clinical Sciences in Lund, Oncology and Pathology, Lund University, Lund, Sweden; 4 Department of Clinical Sciences Lund, Surgery, Lund University, Skåne University Hospital, Medicon Village 406, 223 81 Lund, Sweden; 5 Department of Health Sciences, Lund University, Lund, Sweden

**Keywords:** Breast neoplasm, Breast cancer treatment, Complementary therapies, Exercise, Lymphoedema, Quality of life, Psychosocial, Rehabilitation, Systematic review, Yoga

## Abstract

**Background:**

Breast cancer (BC) is the most common type of cancer in women worldwide. Post-treatment, patients suffer from side effects and have various rehabilitation needs, which means that individualization is fundamental for optimal rehabilitation. This systematic review (SR) of SRs aims to evaluate the current evidence on rehabilitation interventions in female patients following BC treatment.

**Methods:**

Full-text SRs published in English from 2009 were searched in Embase, PubMed, Cinahl Complete, PsycINFO, AMED, SCOPUS, and Cochrane Library. Inclusion criteria: SRs of randomized or non-randomized controlled trials investigating the effects of rehabilitation interventions in women following BC treatment. All outcomes were considered. Methodological quality was evaluated using the AMSTAR 2 tool and interrater agreement was evaluated. Out of 1269 citations retrieved, 37 SRs were included.

**Results:**

Five rehabilitation areas were identified: exercise and physical activity (PA), complementary and alternative medicine (CAM), yoga, lymphoedema treatment, and psychosocial interventions. The most solid evidence was found in exercise/PA and yoga. Exercise interventions improved outcomes such as shoulder mobility, lymphoedema, pain, fatigue and quality of life (QoL). Effects of yoga were shown on QoL, anxiety, depression, sleep disturbance, fatigue and gastrointestinal symptoms. The effect of CAM was shown on nausea, pain, fatigue, anger and anxiety but these results need to be interpreted with caution because of low methodological quality in included studies in the SRs. Among the lymphoedema treatments, positive effects were seen for resistance training on volume reduction and muscle strength and psychosocial interventions such as cognitive behavioural therapy had positive effects on QoL, anxiety, depression and mood disturbance.

**Conclusions:**

This SR of SRs show solid positive effects of exercise/PA and yoga for women following BC treatment, and provides extended knowledge of the effects of CAM, yoga, lymphoedema treatment and psychosocial interventions. It is evident that more than one intervention could have positive effects on a specific symptom and that the effects depend not only on intervention type but also on how and when the intervention is provided. The results can be used as a foundation for individualized rehabilitation and aid health care professionals in meeting patients’ individual needs and preferences.

**Trial registration:**

PROSPERO (CRD42017060912).

**Electronic supplementary material:**

The online version of this article (10.1186/s12885-019-5648-7) contains supplementary material, which is available to authorized users.

## Background

Today breast cancer (BC) is the most common type of cancer in women worldwide [1]. At the same time, developments in diagnostics, treatment and care have resulted in an increased survival rate, which sets new challenges for the health care system regarding how to support patients to achieve optimal rehabilitation. A complicating factor is the heterogeneous rehabilitation needs of women treated for BC. Commonly reported consequences of the disease or treatments are pain [2–4], lymphoedema [5], fatigue [6] and depression [7]. In addition, reduced health-related quality of life (HRQoL) and psychosocial consequences such as reduced social contacts [8] and psychological distress [9] have been reported, as well as difficulty in resuming functional activity and life roles [10], and in unmet information needs [11]. Consequently, patients with BC may have physical, psychological, social and existential rehabilitation needs [12], and more research is needed to identify the optimal way to support them in their new life situation. To optimize rehabilitation, an individualized approach identifying each patient’s specific needs is warranted [13]. However, how this should be done in terms of “who needs what and when” is rarely addressed in either research or clinical practice.

Research often addresses the effects of one specific rehabilitation intervention related to one or a few specific outcomes and provides an enhanced understanding of separate interventions. However, based on the complexity of patients’ rehabilitation needs, such knowledge is often insufficient when it comes to aiding clinicians to promote individualized rehabilitation. To establish a systematic way of providing individualized rehabilitation, further research is warranted to bridge the gap between rehabilitation research and clinical practice.

A complicating factor for health care professionals (HCP) struggling to make sense of and evaluate different rehabilitation alternatives and incorporate them into clinical practice is the large amount of available research on rehabilitation following BC treatment. One way to make evidence available to HCPs and clinical decision makers is by providing them with a summary of available evidence through a systematic review (SR) of systematic reviews (SRs) [14]. The purpose of such a review is to identify and assess all published reviews within a certain area and describe their quality, summarize and compare their conclusions and discuss the strength of these. Through such an approach an evidence base for individualized rehabilitation can be developed.

## Methods

### Aim

This SR of SRs aims to evaluate the current evidence on rehabilitation interventions in female patients following BC treatment.

### Literature search and selection

A systematic search and screening procedure was conducted with assistance from a trained public health librarian. The following databases were systematically examined: Embase, PubMed, Cinahl Complete, PsycINFO, AMED, SCOPUS, and Cochrane Library. The first search was performed in June 2016 and was repeated in October 2017. Several database-adapted MeSH terms were utilized in the initial search, including: “breast cancer”, “breast cancer surgery”, “rehabilitation”, “therapy”, “systematic review” and “meta-analysis”, and the search terms were modified according to the specific vocabulary map of each database (see Additional file 1).

The removal of duplicates and the initial screening of titles and abstracts was performed in EndNote (Clarivate Analytics, Philadelphia, PA, USA). Two of the authors (U.O.M., M.M.) independently reviewed the titles and abstracts. SRs were included if meeting the following inclusion criteria according to the PICO framework, where the population was adult (≥18 years) women who had undergone BC treatment; the intervention, SRs on the effects of single or combined rehabilitation interventions; and the comparison consisted of SRs of randomized controlled trials (RCTs) and non-randomized controlled trials (CTs) including all types of non-randomized trials with a predefined control group. Finally, regarding outcome, all outcomes were considered.

Full-text SRs in the English language published from 2009 in peer-review journals were included. SRs that did not present data separately for BC, were of critically low quality [15] and SRs with fewer than four included studies were excluded. The potential SRs were read in full text by U.O.M., I.B. and M.M. Inclusion and exclusion of SRs were discussed until discrepancies were resolved by consensus among all authors. A protocol for this SR of SRs was registered in PROSPERO (CRD42017060912).

### Quality assessment

The methodological quality and risk of bias in included SRs was evaluated using the AMSTAR 2 tool [15], which is a critical appraisal tool for SRs that include randomised and non-randomised studies of healthcare interventions (NRSI). The instrument consist of 16 items from which five items were considered critical in this SR (item 4; comprehensiveness in literature search, item 9; assessment of risk of bias in individual studies, item 11; appropriateness of methods for statistical combination of results, item 13; account for risk of bias in individual studies when interpreting/discussion results and item 14; explanation for and discussion of heterogeneity). According to AMSTAR 2 [15] multiple non-critical weaknesses may diminish confidence in the review and therefore we chose to move the overall appraisal down from moderate to low confidence if seven or more non-critical weaknesses were found. Quality was rated as high, moderate, low and critically low (excluded) according to the quality rating confidence levels [15]. For quality rating criteria see Table 1 and for quality grading of the included SRs see Table 2.

**Table 1:** Definition of quality rating criteria

Quality rating	Definition
High	No critical flaw and maximum one non-critical weakness
Moderate	No critical flaw and 2–6 non-critical weakness
Low	One critical flaw with or without non-critical weaknesses or 7 or more non-critical weaknesses
Critically low	More than one critical flaw with or without non-critical weaknesses

**Table 2:** AMSTAR 2 quality assessment and interrater agreement

Author (ref number)	Item 1	Item 2	Item 3	Item 4 ^a^	Item 5	Item 6	Item 7	Item 8	Item 9 ^a, b^	Item 10	Item 11^a,b^	Item 12	Item 13^a^	Item 14^a^	Item 15	Item 16	AMSTAR 2 rating
Bluethmann et al., 2015 [16]	No	No	No	Partial yes	No	Yes	No	No	No	No	Yes	Yes	Yes	Yes	Yes	Yes	Low
Chan et al., 2010 [17]	No	No	Yes	Partial yes	No	Yes	Yes	Yes	Partial yes	No	N/A	N/A	Yes	No	N/A	Yes	Low
Chao et al., 2009 [18]	No	No	No	Partial yes	Yes	No	No	Partial yes	Partial yes/no	No	N/A	N/A	Yes	No	N/A	No	Low
Cheema et al., 2014 [19]	No	No	No	Partial yes	Yes	Yes	No	Partial yes	Partial yes	No	Yes	No	No	Yes	Yes	Yes	Low
Cramer et al., 2012 [20]	Yes	No	No	Partial yes	Yes	Yes	No	Yes	Yes	No	Yes	No	Yes	Yes	N/A	Yes	Moderate
Cramer et al., 2017 [21]	Yes	Yes	No	Yes	Yes	Yes	Yes	Yes	Yes	No	Yes	Yes	Yes	Yes	Yes	Yes	Moderate
De Groef et al., 2015 [22]	No	No	Yes	Partial yes	Yes	No	No	Yes	Partial yes	No	N/A	N/A	Yes	Yes	N/A	Yes	Moderate
Devoogdt et al., 2010 [23]	No	No	No	Partial yes	No	No	No	Partial yes	Partial yes/No	No	N/A	N/A	Yes	No	No	No	Low
Duijts et al., 2011 [24]	No	No	Yes	Partial yes	No	Yes	No	No	No	No	Yes	No	Yes	Yes	Yes	Yes	Low
Ezzo et al., 2015 [25]	Yes	Yes	Yes	Partial yes	Yes	Yes	Yes	Yes	Yes	No	Yes	No	Yes	Yes	No	Yes	Moderate
Fors et al., 2011 [26]	No	No	No	No	Yes	No	No	Partial yes	Partial yes	No	N/A	N/A	Yes	Yes	N/A	No	Low
Huang et al., 2013 [27]	No	No	No	Partial yes	No	Yes	No	Partial yes	Partial yes	No	Yes	No	Yes	Yes	No	Yes	Low
Huang et al., 2016 [28]	Yes	No	No	Partial yes	No	Yes	No	Partial yes	Yes/Partial yes	No	Yes/No	No	Yes	Yes	N/A	Yes	Moderate
Jassim et al., 2015 [29]	Yes	Yes	No	Yes	Yes	Yes	Yes	Yes	Yes	Yes	Yes	Yes	Yes	Yes	Yes	Yes	High
Johanssen et al., 2013 [30]	No	No	Yes	Partial yes	Yes	No	No	No	Partial yes/No	No	Yes/No	Yes	Yes	Yes	Yes	Yes	Low
Juvet et al., 2017 [31]	No	No	No	Partial yes	Yes	Yes	No	Yes	Partial yes	No	Yes	No	Yes	Yes	No	Yes	Low
Keilani et al., 2016 [32]	No	No	No	Partial yes	Yes	No	No	Partial yes	No	No	N/A	N/A	Yes	Yes	N/A	Yes	Low
Lee et al., 2010 [33]	Yes	No	No	Partial yes	Yes	Yes	No	Yes	Partial yes/No	No	Yes/No	No	Yes	Yes	No	No	Low
Lee et al., 2016 [34]	Yes	No	No	Partial yes	Yes	Yes	No	Partial yes	Partial yes	No	Yes	No	Yes	No	No	No	Low
Matsuda et al., 2014 [35]	Yes	No	No	Partial yes	No	No	No	No	Partial yes	No	Yes	No	Yes	Yes	No	No	Low
Matthews et al., 2017 [36]	No	Partial yes	No	Partial yes	Yes	No	No	No	No/No	No	Yes/No	Yes	Yes	Yes	Yes	Yes	Low
McNeely et al., 2010 [37]	Yes	Yes	No	Yes	Yes	Yes	Yes	Partial yes	Partial yes	Yes	Yes	Yes	Yes	Yes	No	Yes	Moderate
Meneses-Echavez et al., 2015 [38]	Yes	Yes	No	Partial yes	Yes	Yes	No	Partial yes	Partial yes	No	Yes	Yes	No	Yes	Yes	Yes	Low
Omar et al., 2012 [39]	Yes	No	Yes	Partial yes	No	No	No	Partial yes	Partial yes/No	No	N/A	N/A	Yes	Yes	N/A	Yes	Moderate
Pan et al., 2014 [40]	No	No	No	Partial yes	No	No	No	Partial yes	Yes	No	Yes	No	Yes	Yes	No	Yes	Low
Pan et al., 2015 [41]	Yes	No	No	Partial yes	Yes	Yes	No	Yes	Yes	No	Yes	No	Yes	Yes	Yes	Yes	Moderate
Pan et al., 2017 [42]	Yes	No	No	Partial yes	Yes	No	No	Yes	Yes	No	Yes	No	Yes	Yes	No	Yes	Low
Rogan et al., 2016 [43]	Yes	Yes	No	Partial yes	Yes	Yes	No	Partial yes	Yes/No	No	Yes/Yes	Yes	Yes	Yes	Yes	Yes	Moderate
Shao et al., 2017 [44]	Yes	No	Yes	Partial yes	Yes	Yes	No	Partial yes	Yes	No	Yes	No	Yes	Yes	No	Yes	Moderate
Short et al., 2013 [45]	Yes	No	No	Partial yes	No	Yes	No	Partial yes	Partial yes	No	N/A	N/A	Yes	Yes	N/A	No	Moderate
Singh Paramanandam et al., 2014 [46]	Yes	Yes	Yes	Partial yes	No	No	No	Yes	Partial yes	No	Yes	No	Yes	Yes	No	No	Low
Stuiver et al., 2015 [47]	Yes	Yes	No	Partial yes	Yes	Yes	Yes	Yes	Yes	Yes	Yes	Yes	Yes	Yes	N/A	Yes	High
Xiao et al., 2017 [48]	Yes	No	Yes	Partial yes	No	Yes	No	Partial yes	Partial yes	No	Yes	Yes	Yes	Yes	Yes	No	Moderate
Yan et al., 2014 [49]	Yes	No	Yes	Partial yes	No	Yes	No	Partial yes	Partial yes	No	Yes	No	Yes	Yes	N/A	Yes	Moderate
Zeng et al., 2014 [50]	No	No	No	Partial yes	Yes	No	No	Partial yes	Yes/No	No	Yes/No	Yes	Yes	Yes	No	Yes	Low
Zhang et al., 2012 [51]	Yes	No	Yes	Partial yes	No	Yes	No	Partial yes	Partial yes	No	Yes	No	Yes	Yes	N/A	Yes	Moderate
Zhu et al., 2016 [52]	Yes	No	No	Partial yes	No	Yes	No	Partial yes	Partial yes	No	Yes	Yes	No	Yes	Yes	Yes	Low
Kappa coefficient^C^	0.83	0.88	0.73	0.66	0.64	0.88	1.0	0.56	0.60/0.93	0.79	0.86/0.81	0.71	0.30	0.28	0.74	0.85	

*Item 1*: Did the research questions and inclusion criteria for the review include the components of PICO? *Item 2*: Did the report of the review contain an explicit statement that the review methods were established prior to the conduct of the review and did the report justify any significant deviations from the protocol? *Item 3*: Did the review authors explain their selection of the study designs for inclusion in the review? *Item 4*: Did the review authors use a comprehensive literature search strategy? *Item 5*: Did the review authors perform study selection in duplicate? *Item 6*: Did the review authors perform data extraction in duplicate? *Item 7*: Did the review authors provide a list of excluded studies and justify the exclusions? *Item 8*: Did the review authors describe the included studies in adequate detail? *Item 9:* Did the review authors use a satisfactory technique for assessing the risk of bias (RoB) in individual studies that were included in the review? (RCT/NRSI) *Item 10*: Did the review authors report on the sources of funding for the studies included in the review? *Item 11*: If meta-analysis was performed did the review authors use appropriate methods for statistical combination of results? (RCT/NRSI) *Item 12*: If meta-analysis was performed, did the review authors assess the potential impact of RoB in individual studies on the results of the meta-analysis or other evidence synthesis? *Item 13*: Did the review authors account for RoB in individual studies when interpreting/ discussing the results of the review? *Item 14*: Did the review authors provide a satisfactory explanation for, and discussion of, any heterogeneity observed in the results of the review? *Item 15*: If they performed quantitative synthesis did the review authors carry out an adequate investigation of publication bias (small study bias) and discuss its likely impact on the results of the review? *Item 16*: Did the review authors report any potential sources of conflict of interest, including any funding they received for conducting the review?

^a^Critical domain, ^b^Includes seperate evaluations of RCT and NRIS. ^C^Interrater agreement. Quality rating: High: No critical flaw and maximun one non-critical weakness, Moderate: No critical flaw and 2–6 non-critical weakness, Low: one critical flaw with or without non-critical weaknesses or 7 or more non-critical weaknesses

To ensure interrater reliability, I.B., M.M. and U.O.M. independently scored three SRs at first and then compared and discussed the evaluations. Thereafter all SRs were independently evaluated by two authors (I.B., M.M. or U.O.M.) and discrepancies were discussed until consensus was reached. Interrater agreement was evaluated using Kappa coefficient in the 16 items. Item 9 and 11 includes separate evaluations of RCT and NRSI. A perfect agreement (k = 0.81–1.00) was shown in 8 items, a substantial agreement (k = 0.61–0.80) in 6 items, a moderate agreement (k = 0.41–0.60) in 2 items and fair agreement (k = 0.21–0.40) in 2 items (Table 2).

### Data extraction

Data extraction from the included SRs was performed and independently verified by I.B. and U.O.M. using a standard data extraction form developed by the review authors. The extraction form included: primary author; year of publication; number of studies included; type of rehabilitation method/intervention; total number and range of included participants, clinical information; aim of the SR; inclusion and exclusion criteria; and outcomes. Discrepancies between the authors were resolved through a mutual decision after discussion.

## Results

**Fig. 1:**
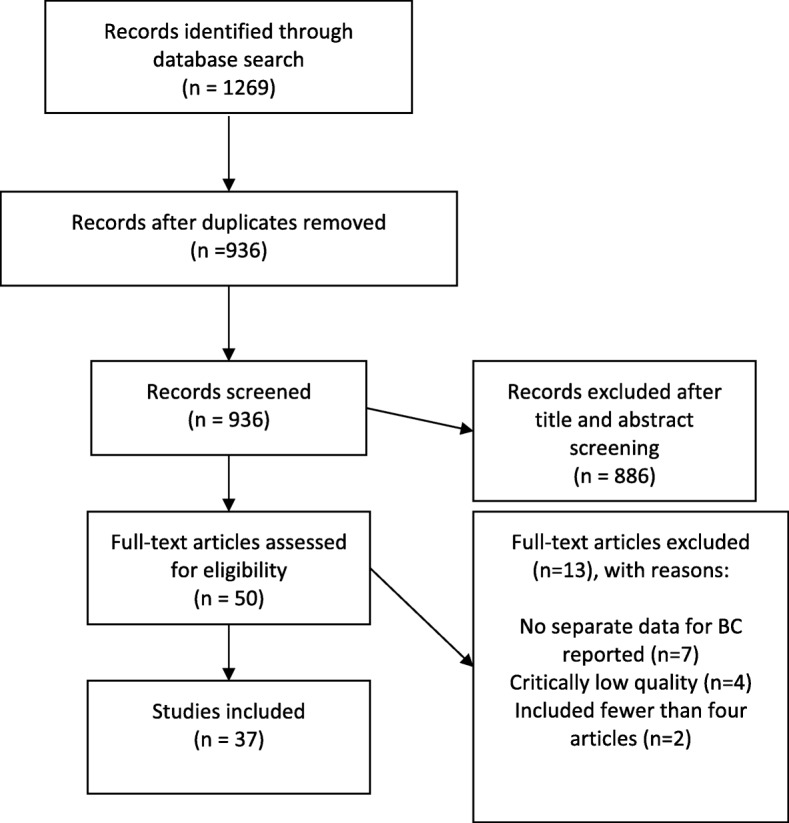
PRISMA flowchart of the identification and inclusion of SRs

The database search yielded 1269 potentially relevant studies, leaving 936 studies after removing duplicates. In total, 886 studies were removed following the review of study titles and abstracts. Of the 50 potentially eligible SRs, 13 were excluded, leaving 37 SRs for inclusion in the review. For details of the identification and inclusion/exclusion of SRs, see PRISMA flow chart (Fig. 1). A summary of the characteristics of the included SRs is presented in Table 3.

**Table 3:** Characteristics of included SRs

	Author, Year (ref. number)	No. and type of included studies	Type of rehabilitation method/intervention	Participants N (range); clinical info	Aim	Inclusion and exclusion criteria	Outcomes
Exercise and physical activity	Juvet et al., 2017 [31]	25 RCTs	Endurance, strength, mobility exercises and coordination.	3418 (41–422). Non-metastatic invasive BC female patients who had undergone surgical procedures followed by chemotherapy or radiotherapy or both. Mostly studies that included early-stage BC patients recruited at least 12 months after diagnosis.	To investigate the efficacy of exercise intervention trials in BC patients during and after adjuvant cancer treatment with respect to HRQoL and with a focus on self-reported physical functioning and fatigue.	Inclusion: 1) RCTs; 2) female BC patients who had undergone surgery; 3) exercise interventions (endurance, strength, mobility exercises and coordination); 4) patient-reported outcomes such as HRQoL outcomes or fatigue; 5) at least 20 participants in each group. Exclusion: low-quality studies, studies with fewer than 20 participants in each group, studies involving patients with metastatic cancer and studies that do not present data separately for BC patients.	Fatigue, physical function.
	Chan et al., 2010 [17]	7 RCTs	Upper limb exercise: posture correction, coordination, resistance machines, free weights, stretching.	429 (27–205). Women undergoing BC surgery with axillary lymph node dissection.	To assess the effectiveness of exercise programmes on shoulder mobility and lymphoedema in postoperative patients with BC having axillary lymph node dissection.	Inclusion: women undergoing BC treatment with axillary lymph node dissection. Various types of exercise programmes: weight training, aerobic and strengthening exercises, stretching and range of motion exercises. Range of shoulder motion, shoulder mobility, arm circumference and arm volume (at least one of these). RCTs published in English. Exclusion: men. No exercise intervention. Patients undergoing sentinel lymph node biopsy.	Range of shoulder motion, shoulder mobility, arm circumference and arm volume.
	Zhu et al., 2016 [52]	33 RCTs	Aerobic, resistance, stretching, yoga, Tai Chi Chuan, dancing.	2659 (19–473). Adults diagnosed with BC, mostly during treatment or post-treatment.	To comprehensively summarize the effects of exercise intervention on BC patients based on the available data from RCTs.	Inclusion: studies that 1) were written in English; 2) had a RCT design, comparing an exercise intervention group with control group (usual care, maintain current activity level, or waiting list); 3) included adults diagnosed with BC; and 4) evaluated the effects of exercise in BC patients. Exclusion: 1) mixed cancer populations, including other types of cancer patients; 2) other types of intervention (exercise intervention combined with diet); and 3) exercise merely focused on upper limb or arm.	QoL, depression, anxiety, fatigue, muscle strength, body composition, physiological markers.
	Meneses-Echavez et al., 2015 [38]	9 RCTs	Supervised exercise interventions defined as “any body movement causing an increase in energy expenditure that involves a planned or structured movement of the body performed in a systematic manner in terms of frequency, intensity, and duration and is designed to maintain or enhance health-related outcomes”.	1156 (22–500). BC survivors (women) stages I-IIIA.	To determine the pooled effects of supervised exercise interventions on cancer-related fatigue in BC survivors, via a meta-analysis of RCTs.	Inclusion: RCTs involving BC without restrictions regarding stage of disease. Supervised exercise interventions. Exclusion: systematic reviews, editorials, cross-sectional studies, case reports and case series studies, non-supervised exercise programmes, Tai Chi, manual therapy (joint mobilization techniques and therapeutic massage) and cognitive behavioral interventions. Studies that compared supervised exercise with pharmacological and surgical treatments.	Primary: fatigue. Secondary: depression; BMI as an indicator of body composition closely related to cancer progression; physical activity levels (minutes per week); QoL including physical, social, emotional and functional wellbeing.
	Zeng et al., 2014 [50]	22 RCTs, 3 CTs	Any type of exercise (aerobic, anaerobic, or a combination of these).	2908 (18–573). Women who had completed active BC treatment.	To determine the effectiveness of exercise interventions on overall QoL and domain-specific QoL in BC survivors.	Inclusion: studies in English or Chinese. Participants were at least 18 years old, had a diagnosis of BC, and had completed active BC treatment. Any type of exercise (aerobic, anaerobic, or a combination) with BC surgery.	QoL outcomes measured by generic, cancer-specific, or cancer site-specific QoL scales.
	Bluethmann et al., 2015 [16]	14 RCTs	Physical activity and behaviour change	2140 (36–500). BC surgery 5 years or less from completion of active cancer treatment. Most participants reported receiving an early cancer diagnosis at Stage I or Stage II of disease. Most studies excluded women diagnosed at Stage IV.	To 1) describe the characteristics of PA behaviour interventions for BC patients, including targeted populations, intervention features, and use of behavior theory; and 2) determine effect size estimates for behavior change from these PA interventions.	Inclusion: 5 years or less from completion of active treatment, including behaviour interventions; interventions targeting moderate to vigorous physical activity (MVPA) but not requiring access to exercise facilities or equipment.	Mean minutes of moderate to vigorous physical activity or mean hours of Metabolic Equivalent per week.
	Duijts et al., 2011 [24]	56 RCTs	Behavioral techniques and/or physical exercise	7164 (24–558). Stages 0-IV. Mostly non-metastatic BC patients.	To evaluate the effect of behavioral techniques and physical exercise on psychosocial functioning and HRQoL outcomes in BC patients and survivors.	Inclusion: RCTs that addressed the effect of behavioral techniques or physical exercise on psychosocial functioning and HRQoL outcomes.	Psychosocial functioning, HRQoL, fatigue, depression, anxiety, body image, stress.
	De Groef et al., 2015 [22]	18 RCTs	Passive mobilization, manual stretching, myofascial therapy, active exercises	1105 (61–439). Women who had undergone surgery for BC with axillary lymph node dissection and/or sentinelnode biopsy and/or modified radical mastectomy.	To investigate the effectiveness of four different physical therapy modalities on postoperative upper limb pain and impaired ROM after BC treatment.	Inclusion: women who had undergone surgery for BC. The physical therapy programme had to be started within 6 weeks of surgery.	Primary: pain and/or ROM of the shoulder. Secondary: e.g. decreased strength, arm lymphoedema, limitations in activities of daily living, QoL, wound drainage volume, seroma formation, punction volume.
	McNeely et al., 2010 [37]	24 RCTs	Exercise for upper limb dysfunction: 1) active or active-assisted ROM exercises; 2) passive ROM/manual stretching exercises; 3) stretching exercises (including formal exercise interventions such as yoga and Tai Chi Chuan); 4) strengthening or resistance exercises.	2132 (21–344). Women who had undergone surgical removal of breast tumour (e.g. radical mastectomy, modified radical mastectomy, local wide excision and lumpectomy); axillary lymph node dissection (AND)/SNB/sentinel node dissection.	To examine RCTs for evidence of effectiveness of exercise interventions to prevent, minimize and/or improve upper limb dysfunction due to BC treatment.	Inclusion: RCTs; participants: 1) confirmed BC diagnosis; 2) surgical removal of breast tumour (e.g. radical mastectomy, modified radical mastectomy, local wide excision and lumpectomy);3) axillary lymph node dissection (AND)/SNB/sentinel node dissection; 4) adults: 17 years and older. Exclusion: cancer other than BC except BC subgroup.	Primary: upper extremity ROM, muscular strength, lymphoedema, pain. Secondary: upper extremity/shoulder function and QoL. Early post-operative complications (adverse events).
	Short et al., 2013 [45]	10 RCTs	Behavioural change interventions for physical activity	1299 (36–404). Adult post-treatment (not including hormone therapy) BC survivors.	To examine the efficacy of behavioural interventions for promoting physical activity among post-treatment BC survivors.	Inclusion: studies that 1) examined the efficacy of at least one behaviour modification intervention designed to promote physical activity (i.e. aerobic activity and/or resistance training) among adult post-treatment (not including hormone therapy) BC patients; 2) included either self-reported or objectively assessed physical activity behaviour change as a study outcome; and 3) used an individual or cluster randomized controlled design. Exclusion: studies that 1) were published in a language other than English; 2) reported the efficacy of a physical activity intervention that did not involve behaviour change techniques (for example, a supervised exercise programme with no intervention component targeting increased knowledge or skills); 3) included mixed samples of cancer survivors (including BC survivors) and did not report intervention effects specifically by cancer type; 4) included BC survivors still undergoing active treatment (defined as: surgery, chemotherapy and radiotherapy); or 5) were available as a conference abstract only.	Physical activity (self-reported, using a pedometer or accelerometer).
Complementary and alternative medicine	Chao et al., 2009 [18]	26 RCTs	Acupoint stimulation	1548 (5–160). Adults with BC at any stage and undergoing treatments (surgery, radiotherapy, chemotherapy, hormonal therapy, or palliative treatment for metastatic BC), experiencing treatment-induced adverse events.	To scrutinize the evidence of using acupuncture point stimulation by any modality for managing adverse events related to anticancer therapies in patients with BC.	Inclusion: 1) study design: clinical trials including RCTs, CCTs, or single-group studies; 2) participants: adults who were diagnosed with BC at any stage and undergoing treatments such as surgery, radiotherapy, chemotherapy, hormonal therapy, or palliative treatment for metastatic BC, and experiencing treatment-induced adverse events; 3) intervention: stimulation of acupuncture points by any modality; 4) outcome measures: at least one clinically related outcome variable such as symptom scores; as well as condition-specific outcomes or generic health status outcomes. Exclusion: animal studies, case reports and anecdotal evidence, qualitative studies or descriptive surveys, and reports that were available only in abstract form; as well as diagnosis other than BC unless separate data were available for the BC subgroup.	Chemotherapy-induced nausea vomiting, vasomotor syndrome, post-operational pain, radiotherapy or chemotherapy-induced leukopenia, AI-induced arthralgia, and BC-related lymphoedema.
	Pan et al., 2014 [40]	18 RCTs	Massage	950 (14–134). Female participants aged 18 years or older, history of BC, receiving active BC treatment. Mostly stage I-III.	To assess the efficacy of massage on treatment-related side effects and QoL in patients with BC.	Inclusion: participants: 1) aged 18 years or older and 2) with a history of BC and 3) receiving active BC treatments; studies: 4) RCTs which examined the effects of massage on treatment-related symptoms (pain, fatigue, sleep disturbances, gastrointestinal symptoms and/or negative mood).	Depression, anger, anxiety, fatigue, pain, upper limb lymphoedema, cortisol, HRQoL.
	Lee et al., 2016 [34]	23 RCTs	Acupoint stimulation, massage therapy and expressive writing.	2346 (12–507). Female BC patients without any restrictions on age, race, status of severity, duration of cancer or clinical status. Mostly stage 0-IIIa.	To determine the effects on QoL, negative emotions and disease-related symptoms among women with BC.	Inclusion: all women with BC, without any restrictions on age, race, status of severity, and duration of cancer. There were no restrictions regarding patients’ clinical status (e.g. active treatment or post-treatment). Exclusion: studies involving interventions for people with a range of conditions (including people with cancers other than BC).	Primary: QoL and pain. Secondary: anxiety, depression, fatigue, sleep quality.
	Lee et al., 2010 [33]	3 RCTs, 4 CTs	Tai Chi	201 (30–78). BC patients stage I-IV.	To critically evaluate the clinical trial evidence for or against the effectiveness of Tai Chi for providing supportive care in patients with BC.	Inclusion: prospective CCTs, Tai Chi alone or combined with other treatments. Exclusion: trials with designs that did not allow for an evaluation of the effectiveness of the intervention (e.g. by using treatments of unproven efficacy in the control group or comparing two different forms of Tai Chi).	Primary: symptoms. Secondary: survival rate, QoL.
	Pan et al., 2015 [41]	9 RCTs	Tai Chi	273 (16–73). Female participants aged 18 years or older, history of BC, received active BC treatment. Mostly stage I-III.	To evaluate measures of pathology, physical activity, and overall wellbeing from the available RCTs.	Inclusion: participants: 1) aged 18 years or older; 2) had a history of BC; and 3) received active BC treatment; studies: 4) examined the effects of Tai Chi Chuan on psychological symptoms (stress, anxiety, and/or depression), treatment-related symptoms (e.g. pain and/or fatigue), or regulation of inflammatory responses and other biomarkers.	Pain, Interleukin 6, Insuline-like Growth Factor 1, Handgrip Dynamometer, Flexibility (degrees), BMI, physical, social or emotional well-being, general HRQoL.
	Yan et al., 2014 [49]	9 RCTs	Tai Chi	407 (19–134). BC survivors.	To assess the effects of Tai Chi on QoL and other important clinical outcomes in BC survivors.	Inclusion: 1) participants: patients with diagnosed BC; 2) intervention: Tai Chi or TaiJi Chuan exercise with or without other treatments; 3) comparison: other treatments including standard support therapy, psychosocial support therapy, usual health care, or other forms of exercise.	Primary: QoL. Secondary: BMI, bone mineral density, muscle strength.
Yoga	Pan et al., 2017 [42]	16 RCTs	Yoga	930 (18–128). Patients with stage 0-III BC and patients with cancer of varying stages.	To determine whether yoga as a complementary and alternative medicine was associated with enhanced health and treatment-related side effects in patients with BC, and examine whether yoga practice provides any measurable benefit, both physically and psychologically, for women with BC.	Inclusion: female participants 1) aged 18 years or older; 2) with a history of BC; and 3) receiving active BC treatments. Studies: 1) RCTs if they examined the effects of yoga practices on psychological symptoms (stress, anxiety and/or depression) and treatment-related symptoms (pain, fatigue, sleep disturbances and/or gastrointestinal symptoms); 2) different control groups in RCTs examining clinical characteristics in parallel to yoga therapy.	Depression, anxiety, physical wellbeing, overall HRQoL, fatigue, sleep quality, gastrointestinal symptoms, and pain.
	Cramer et al., 2012 [20]	12 RCTs	Yoga	742 (19–168). BC patients and survivors.	To assess and meta-analyse the evidence for effects of yoga on HRQoL and psychological health in BC patients and survivors.	Inclusion: 1) RCTs if published as full paper; 2) studies of adult (> 18 years) patients with a history of BC; 3) studies comparing yoga with no treatment or any active treatment. Studies were eligible if they assessed HRQoL or wellbeing (global HRQoL, mental, physical, functional, social, and/or spiritual wellbeing) and/or psychological health (depression, anxiety, perceived stress, and/or psychological distress). If available, safety data served as secondary outcome measures. Exclusion: if yoga was not the main intervention but part of a multimodal intervention.	Primary: short and long-term effect on HRQoL or wellbeing (global HRQoL, mental, physical, functional, social and/or spiritual wellbeing) and/or psychological health (depression, anxiety, perceived stress and/or psychological distress, mood). Secondary: safety data, i.e. reported adverse events.
	Zhang et al., 2012 [51]	6 RCTs	Yoga	382 (18–164). Women with non-metastatic or metastatic BC.	To evaluate the effects of yoga on psychologic function and QoL in women with BC.	Inclusion: 1) RCTs, comparing yoga alone or a yoga-based intervention with a control group receiving no intervention, for psychological functioning and QoL in women with BC; 2) studies that examined yoga as a main intervention. Exclusion: 1) studies that included yoga as part of a larger intervention programme (e.g. mindfulness stress-reduction training), and those that did not provide findings specific to yoga.	Anxiety, depression, distress, perceived stress, fatigue, sleep and QoL.
	Cramer et al., 2017 [21]	24 RCTs	Yoga	2166 (18–309). Women with non-metastatic BC (23 RCTs) and non-metastatic and metastatic carcinoma (1 RCT).	To assess effects of yoga on HRQoL, mental health and cancer-related symptoms.	Inclusion: RCTS assessing effects of yoga in women with BC (histologically confirmed diagnosis of non-metastatic or metastatic carcinoma) who were undergoing treatment or had completed treatment, or both. Exclusion: studies not providing measures of dispersion.	Primary: HRQoL, depression, anxiety, fatigue and sleep disturbances. Secondary: safety of the intervention, assessed as number of women with adverse events and number of women with severe adverse events.
	Keilani et al., 2016 [32]	9 RCTs	Resistance exercise	957 (17–242). BC patients with or at risk of secondary lymphoedema (changes in BC survivors with pre-existing lymphoedema, the volume of the upper extremities in BC survivors at risk of lymphoedema, or included BC survivors both with or without pre-existing lymphoedema).	To investigate firstly, whether resistance exercise increases the risk/causes of development of BCRL and, secondly, whether patients with BCRL deteriorate, improve, or stay the same with resistance exercise.	Inclusion: prospective randomized controlled studies investigating the effect of a resistance exercise intervention on development of secondary lymphoedema in BC survivors.	Lymphoedema status, physical performance and function, body composition, QoL.
	Singh Paramanandam et al., 2014 [46]	11 RCTs	Weight training or resistance exercises	1091 (40–204). Women of any age who had lymphoedema or were at risk of developing lymphoedema during or following BC treatment (modified radical mastectomy or breast conservationsurgery along with various axillary node management).	Research questions: 1) Is weight training exercise safe for women with lymphoedema or at risk of lymphoedema after BC? 2) Does weight training exercise improve muscle strength, QoL and BMI in this population?	Inclusion: 1) RTs conducted after 2001; 2) women with BC diagnosis with or at risk of developing lymphoedema; 3) weight training exercises.	Lymphoedema onset or exacerbation, limb strength, QoL, BMI.
Lymphoedema treatment	Cheema et al., 2014 [19]	15 RCTs	Progressive resistance training	1652 (21–232). Women surgically treated for primary tumour of the breast. Completion of all BC-related treatments (except hormonal therapy) or initiation of chemotherapy treatment for BC. Lymph node dissection (or SNB) and/or clinical diagnosis of lymphoedema by clinician.	To assess the safety and efficacy of progressive resistance training in BC.	Inclusion: 1) participants: women surgically treated for primary tumour of the breast; 2) intervention: PRT interventions at least 6 weeks in duration; 3) studies: studies including flexibility training plus PRT (PRT involving aspects of flexibility training, i.e. loaded movements throughout a complete ROM). Where multiple PRT interventions were tested, higher-intensity regimens were prioritized over lower-intensity regimens; published in English. Exclusion: Intervention studies that prescribed aerobic training plus PRT, unless a comparison group undertook the same dosage of aerobic training in isolation.	Primary: safety: 1) cases of BCRL incidence or exacerbation during the trial; 2) arm volume outcomes; and 3) BCRL symptom severity comparison between the treatment and the control group.Secondary: efficacy: 1) upper body strength; 2) lower body strength; 3) comparison of HRQoL after intervention (post-treatment) between the treatment and the control group.
	Huang et al., 2013 [27]	10 RCTs	MLD	566 (24–158). Women who had undergone mastectomy with axillary lymph node dissection.	To evaluate the effectiveness of MLD in the prevention and treatment of BCRL.	Inclusion: 1) women who had undergone mastectomy with axillary lymph node dissection; inclusion criteria also concerned: 2) the MLD technique used; 3) the compression strategy used; 4) the definition of lymphoedema; and 5) evaluation of lymphoedema severity. Exclusion: 1) patients who had not received axillary lymph node dissection (e.g. in studies in which only sentinel node sampling was used); 2) studies in which the clinical outcomes had not been clearly stated; and 3) duplicate reporting of patient cohorts.	Incidence of lymphoedema, reduction in lymphoedema volume.
	Devoogdt et al., 2010 [23]	10 RCTs, 5 CTs	Combined Physical Therapy, Intermittent Pneumatic Compression, arm elevation.	656 (14–80). Patients with arm lymphoedema, in the majority developed after axillary dissection for BC.	To review the available literature on different physical treatment modalities for lymphoedema.	Inclusion: RCTs, pseudo-randomised controlled trials and non-randomised experimental trials investigating the effectiveness of Combined Physical Therapy and its different parts, of Intermittent Pneumatic Compression and of arm elevation were included.	Arm volume, shoulder mobility, musclestrength, subjective symptoms, tissue elasticity, skinfold thickness and quality of life.
	Omar et al., 2012 [39]	8 RCTs	Low-level laser therapy	230 (10–64). Women with unilateral lymphoedema secondary to BC surgery and/or radiotherapy.	To review the effect of low-level laser therapy on management of BCRL.	Inclusion: RCTs and uncontrolled trials. Women (greater than 18 years old) with unilateral lymphoedema secondary to BC surgery and/or radiotherapy. Exclusion: recurrent malignant disease.	Volume and/or circumference.
	Shao et al., 2017 [44]	4 RCTs	MLD	234 (41–88). Patients undergoing treatment of breast carcinoma and having lymphoedema.	To compare the effectiveness of MLD for the management of BCRL.	Inclusion: patients undergoing treatment of breast carcinoma and having lymphoedema defined as a minimum of 10% or 2 cm or 150 mL volume difference between the affected and the unaffected arm.	Primary: volume reduction. Secondary: improvement of symptoms and arm function.
	Ezzo et al., 2015 [25]	6 RCTs	MLD	426 (52–95). Women diagnosed with BCRL in any body area (i.e. arm, hand, trunk).	To assess the efficacy and safety of MLD in treating BCRL.	Inclusion: randomized or quasi-randomized (i.e. allocated by alternate assignment, date of birth, etc) trials in any language.	Primary: 1) volumetric changes in arm, hand, breast or trunk; 2) adverse events. Secondary: functional measures, subjective sensations, QoL and other psychosocial outcomes, cost of care, any other outcome reported by the trial.
	Rogan et al., 2016 [43]	32 RCTs	Lymphatic drainage or lymph tape or compression bandage or sleeve or intermittent pneumatic compression or exercise.	1337 (14–141). Female BC patients with lymphoedema.	To study the effects of compression (bandages) and active exercise during the intensive phase of therapy on the reduction of lymphoedema in BC patients.	Inclusion: 1) RCTs; 2) adequate statistics for a meta-analysis; 3) written in English or German. Exclusion: 1) effects of drugs, hormonal therapy, or radiation and surgical procedures; 2) studies in children; 3) non-BCs, lower-extremity oedema; 4) impact on fatigue only; 5) diet, or sexually transmitted diseases; 6) cost analysis only; and 7) non-carcinogenic syndromes or 8) prevention of BC.	Volume or oedema reduction.
	Stuiver et al., 2015 [47]	10 RCTs	Conservative lymphatic interventions	1205 (48–205). Participants of both sexes and all ages at risk of developing lymphoedema in the upper limb after treatment for BC (surgical treatment for BC with axillary lymph node dissection, SNB or axillary sampling, with or without radiotherapy to the axilla or the supraclavicular fossa or both, or radiotherapy alone).	To assess the effects of conservative (non-surgical and non-pharmacological) interventions for preventing clinically detectable upper limb lymphoedema after BC treatment.	Inclusion: 1) studies: RCTs that reported secondary lymphoedema as the primary outcome, and that compared a conservative intervention to either usual care, placebo intervention, or some other intervention. No language or publication date restrictions were imposed. We only considered research published in peer-reviewed scientific journals; 2) participants: persons of both sexes and all ages at risk of developing lymphoedema in the upper limb after treatment for BC; 3) intervention: surgical treatment for BC with axillary lymph node dissection, SNB or axillary sampling, with or without radiotherapy to the axilla or the supraclavicular fossa or both; or radiotherapy alone. Exclusion: persons diagnosed with lymphoedema/cancer recurrence.	Primary: lymphoedema (circumference measurements, water displacement methods, bioimpedance measurements, laser scanning, perimetry and dual-energy X-ray absorptiometry scanning), time to event. Secondary: infection, ROM of the upper limb, activities of daily living, pain, HRQoL.
Psychosocial interventions	Fors et al., 2011 [26]	18 RCTs	Psychoeducational information	3272 (27–303). Women with BC undergoing surgery and adjuvant treatment.	To determine the effectiveness of psychoeducation, CBT and social support interventions used in the rehabilitation of BC patients.	Inclusion: RCTs studying the effect of psychosocial interventions on BC rehabilitation in ≥20 female BC patients after undergoing surgery and adjuvant treatment in groups. Exclusion: studies with metastatic BC patients and studies including other cancer types; data not presented separately for BC; low-quality studies; < 20 participants in each group.	QoL, fatigue, mood, health behaviours and social function.
	Matsuda et al., 2014 [35]	8 RCTs	Psychoeducation and psychosocial support	1159 (49–256). Early-stage BC patients.	To evaluate the effectiveness of psychosocial and especially psycho-educational support interventions to improve QoL for early-stage BC patients, with a follow-up of up to 6 months after completing the intervention.	Inclusion: RCTs on BC comparing a group receiving social support with a control group. Exclusion: 1) patients with metastatic or advanced-stage cancer; 2) intervention studies that included exercise as social suport; 3) studies not reporting adequate information on the randomization process and not reporting HRQoL data using a QoL questionnaire.	Global QoL, BC symptoms, emotional wellbeing.
	Johanssen et al., 2013 [30]	16 RCTs, 10 CTs	Patient education, supportive group therapy, relaxation therapies	2193 (8–309). Women with BC, stage 0 to IV, most of whom completed treatment.	To systematically review and quantify the existing research on the effect of psychosocial interventions on pain in BC patients and survivors.	Inclusion: Studies that presented data on a psychosocial interventions, including both baseline and post-intervention measures of pain, and that reported data on BC populations and used a quantitative research approach.	Pain.
	Matthews et al., 2017 [36]	22 RCTs, 10 CTs	CBT, psychoeducational interventions, support groups, counselling, supportive-expressive group therapy, mindfulness-based stress reduction programme, psychosexual intervention, music therapy and progressive music relaxation training	4148 (20–442). Women after BC surgery.	To identify the efficacy of psychosocial interventions for women following BC surgery.	Inclusion: 1) participants: female adult BC survivors following any type of primary BC surgery; 2) intervention: psychological, psychoeducational, and/or psychosocial intervention; 3) studies: written in English; using quantitative methodology; presenting empirical findings. Exclusion: 1) interventions with focus on physical rehabilitation, physiological outcomes, and palliative and/or metastatic BC; 2) research published as a conference abstract or a case study.	Anxiety, depression, QoL, mood disturbance, distess, body image, sleep disturbance, self-esteem, sexual function.
	Huang et al., 2016 [28]	3 RCTs, 5 CTs	Mindfulness-based stress reduction programme	964 (13–336). Women with BC.	To evaluate the benefits of mindfulness-based stress reduction programme on psychological distress among BC survivors.	Inclusion: RCT and before-and-after intervention study comparing mindfulness-based stress reduction programme with standard/usual care in women diagnosed with BC. Outcomes: QoL and psychological domains. Exclusion: mixed cancers; unpublished or duplicate data, insufficient raw data.	Primary: psychological domains such as depression, anxiety, stress. Secondary: effects on QoL.
	Xiao et al., 2017 [48]	13 RCTs	Psychological education, relaxation training, psychological counselling, CBT	966 (−). Women who had been diagnosed with BC and had undergone BC surgery.	To assess the efficacy of individually delivered CBT in improving the depressive symptoms of women with BC.	Inclusion: RCTs comparing individually delivered CBT or CBT-based interventions with a control group receiving no intervention for depression disorders in women after BC surgery.	Depression and anxiety.
	Jassim et al., 2015 [29]	28 RCTs	Cognitive behavioral interventions, psychotherapy counselling and informational and psycho-educational therapy	3940 (14–575). Women witth non-metastatic BC.	To assess the effects of psychological interventions on psychologicalmorbidities, QoL and survival among women with non-metastatic BC.	Inclusion: 1) RCTs comparing any form of psychological or behavioural intervention with a placebo, waiting list controls or an alternative form of psychological intervention; 2) women with a histologically confirmed diagnosis of breast carcinoma of an early non-metastatic stage.	Primary: depression, anxiety, stress and mood disturbance. Secondary: effects on QoL, coping, adjustment and survival.

*Abbreviations:* Body Mass Index (BMI), Breast Cancer (BC), Breast Cancer Related Lymphoedema (BCRL), Cognitive-behavioral therapy (CBT), Controlled trials (CT) = Includes all types of non-randomized trials, Manual Lymphatic Drainage (MLD), Health-Related Quality of Life (HRQoL), Physical Activity (PA), Progressive Resistance Training (PRT), Randomized Controlled Trial (RCT), Range of Motion (ROM), Quality of Life (QoL)

To provide a comprehensive overview within this complex and largely varying rehabilitation area, a broad scope of SRs evaluating the effect of rehabilitation interventions for patients were included. Five rehabilitation areas were identified during the analysis process: exercise and physical activity (PA), complementary and alternative medicine (CAM) interventions, yoga, lymphoedema treatment, and psychosocial interventions.

### Quality assessment of included SRs

Methodological quality of the 37 included SRs were evaluated with the AMSTAR 2 [15] tool and 21 were rated as having low, 14 as having moderate and two as having high methodological quality. Ten SRs within the *exercise and PA* area was evaluated and seven of them were rated as having low [16, 17, 24, 31, 38, 50, 52] and three as having moderate [22, 37, 45] methodological quality. Six SRs within the *CAM intervention* area were evaluated and four of them were rated as having low [18, 33, 34, 40] and two as having moderate [41, 49] methodological quality. In the *yoga intervention* area a total of four SRs were evaluated and one was rated as having low [42] and three as having moderate [20, 21, 51] methodological quality. In the *lymphoedema treatment* area ten SRs were evaluated of which five were rated as having low [19, 23, 27, 32, 46], four as having moderate [25, 39, 43, 44] and one as having high [47] methodological quality. Seven SRs within the *psychosocial intervention* area were evaluated and four were rated as having low [26, 30, 35, 36], two as having moderate [28, 48] and one as having high [29] methodological quality.

### Exercise and PA

Overall, exercise and PA interventions were found to be beneficial [52], safe and feasible [38] and positive effects were shown on several outcomes.

#### Upper limb dysfunction

Exercise interventions such as range of motion and aerobic, resistance and stretching exercises were evaluated. Positive effects such as increased shoulder mobility [17, 22, 37] and reduced shoulder pain were shown [22].

#### Fatigue

Mixed exercise programs such as aerobic exercise and resistance training showed significant reductions of fatigue in some [31, 38] but not all [52] SRs. A more pronounced effect was identified with increasing length, duration and frequency of the intervention [38] and when performed after instead of during adjuvant BC treatment [31].

#### Quality of life

Aerobic exercise [38, 50, 52] and resistance training [38, 50] as well as a mix of exercise and PA (e.g. walking) showed positive effects on QoL [52]. Supervised exercise programs seemed to be more effective than home-based programs [38]. Interventions for upper limb dysfunction showed no effect on QoL [37].

#### Behavior change interventions to promote exercise and PA

Behavior change interventions (health education, stress management and psychology-based therapy) included different strategies such as telephone counselling, workshops, group exercise and web-based support. The results varied greatly relative to type of intervention strategies. Positive effects on fatigue, depression, anxiety and stress were demonstrated [24]. Modest positive short-term effects was shown on the amount of PA [16, 45]. A significant increase in PA was demonstrated, mainly in SRs including high supervision/monitoring, but also in SRs with less intense supervision, such as counselling by telephone or e-mail [16].

In *conclusion*, exercise and PA was shown to have positive effects on physical function, pain, fatigue and QoL. Behavioural interventions showed positive effects on e.g. fatigue and may increase the amount of PA. However, the mediators and sustainability of intervention effects are not known.

### CAM interventions

Tai Chi was shown to have positive effects on emotional wellbeing [49] and short-term benefits on upper limb functional mobility [41]. Inconclusive results was shown on overall QoL, psychological variables (e.g. self-esteem, mood) and physical outcome measures (e.g. hand-grip strength, flexibility) [33]. No effects of Thai Chi were seen on body mass index (BMI), bone mineral density and muscle strength [49]. Acupoint stimulation, in particular acupressure on the P6 acupoint, may be beneficial to reduce chemotherapy-induced nausea and vomiting [18] while massage can reduce anger and fatigue [40]. One SR showed positive effects of acupoint stimulation for pain and fatigue, as well as massage on anxiety, and that expressive writing had benefits for QoL [34].

In *conclusion*, CAM may have positive effects on nausea, pain, fatigue, anger, anxiety, upper limb functional mobility and QoL. However, these results should be interpreted with caution because the intervention studies included in the SRs were reported as being built on small studies with low methodological quality.

### Yoga

Yoga had positive effects and significantly improved QoL/HRQoL [20, 21, 42, 51] and was also shown to reduced fatigue, sleep disturbance [21], gastrointestinal symptoms [42], anxiety and depression [20, 42]. Subgroup analyses revealed symptom relief only during active cancer treatment [20] and that yoga had positive effects on anxiety only when it had been practiced for longer than 3 months [42]. When yoga was compared with psychosocial/educational interventions, positive moderate effects were seen in anxiety, depression and fatigue in favour of yoga [21].

In *conclusion*, yoga was suggested to have positive effects on QoL, anxiety, depression, sleep disturbance, fatigue and gastrointestinal symptoms. Duration as well as phase of cancer treatment seemed to be key to a positive outcome.

### Lymphoedema treatment

#### Impact on risk for developing lymphoedema

Progressive resistance exercise therapy and shoulder-mobilizing exercises do not appear to increase the risk of developing lymphedema. Symptoms should be closely monitored and adequately treated if they occur. Shoulder-mobilizing exercise seems to be more beneficial when started earlier, rather than later [47].

#### Impact on lymphoedema

Resistance training seemed to be safe as it did not increase the severity of lymphoedema, and was beneficial in terms of increased upper and lower body muscular strength [19, 32, 46], QoL [19, 46] and maintained BMI [46]. Exercise (yoga, Nordic walking, resistance training) showed positive effects on volume reduction [43]. In the acute phase intermittent pneumatic compression may be beneficial in combination with other therapies to reduce the oedema volume [43]. Sleeves does not seem to reduce the volume, but may prevent additional swelling [43]. Combined physical therapy may be effective for arm oedema reduction [23]. Low-level laser therapy was effective for volume reduction and a dose of 1–2 J/cm^2^ per point, applied to several points covering the fibrotic area, was recommended [39]. The effects of manual lymphatic drainage (MLD) for reducing lymphedema were inconclusive [23, 27]. For example, it was shown that MLD significantely reduced arm volume but might not improve subjective symptoms or arm function [44]. MLD was well tolerated and safe in combination with compression therapy and may benefit women with mild to moderate lymphedema [25]. It was also shown that compression bandages or fitted sleeves appear to be more effective than standard sleeves and that it is important to do regular check-ups for volume status [25].

In *conclusion*, resistance training did not seem to increase the risk of developing lymphoedema or worsen existing lymphoedema. Positive effects on volume reduction and muscular strength were shown. The effect of MLD seems to be related to the severity of the lymphoedema and treatment combinations. Combined physical therapy, low-laser therapy, exercise and sleeves seemed to have positive effects.

### Psychosocial interventions

Cognitive behavioral therapy (CBT) interventions showed positive effects on anxiety, depression [29, 36, 48], QoL [26, 29, 36], mood disturbance [29], body image, sleep disturbance and self-esteem [36]. Individualized CBT sessions may positively impact QoL [29] and depressive symptoms [48]. In early stage BC patients receiving psychosocial support, no effects were shown for QoL [35]. A mindfulness-based stress reduction intervention significantly improved anxiety, depression, stress and overall QoL [28] and patient education programmes had a significant effect on pain reduction [30].

In *conclusion*, psychosocial interventions may have positive effects on anxiety, depression and QoL. However, these results should be interpreted with caution because the intervention studies included in the SRs were built on small, heterogeneous studies with low methodological quality.

## Discussion

This SR synthesizes the existing literature on BC rehabilitation and provides a comprehensive overview of the effects of various rehabilitation interventions that can be used as a foundation for individualized rehabilitation in clinical practice.

Five rehabilitation areas were identified and most of the evidence was found in the areas of exercise and PA, and yoga. Our results are in line with an earlier SR of SRs, which found strong evidence for exercise interventions aiming to improve physical outcomes such as shoulder mobility and reduced lymphoedema [53]. However, our results also show that exercise seems safe and feasible in general and specifically for shoulder pain and mobility. Exercise also showed predominately positive effects on fatigue and QoL. Yoga was found to have positive effects on psychological wellbeing as well as some aspects of physical wellbeing, especially during active cancer treatment. Despite the evidence of positive effects of exercise, it is known that BC patients do not meet the recommended level of PA [54]. PA has an established and potent impact on mortality [55] and is also related to better QoL in patients with cancer [56]. This indicates that there is a great need for HCP to encourage patients to exercise during the cancer trajectory. Even though the optimal type, frequency, intensity and duration of exercise and PA are still unknown it is clearly stated that performing any kind of these interventions may have positive effects and is safe and feasible. HCPs need to focus on supporting patients, not only in exercise and PA, but also in self-care, work and leisure, to increase their confidence and motivation [10].

In line with the previous SR of SRs [53] the present study shows that there is an overemphasis on SRs focusing on physical interventions such as exercise, PA and yoga. Despite the existing evidence related to specific exercise interventions it is clear that one symptom or problem could be treated with a range of interventions. As an example, patients suffering from fatigue could benefit from interventions such as exercise [31, 38], CAM (acupoint stimulation and massage) [34, 40] or yoga [21] depending on the diverse array of etiological origins of fatigue [57] and, also, on the patients’ preferences. Exercise and yoga likewise have shown effects on anxiety, depression and QoL [42, 52]. Anxiety was also reduced by CBT, mindfulness-based stress reduction and massage [28, 34, 36]. This variety of interventions with positive outcomes indicates that it should be possible to optimize rehabilitation through evidence-based interventions. However, to enable this HCP need tools to identify patients’ needs and knowledge, both in how to do this and about available and effective interventions. Therefore, taking the next step from evaluating the effect of narrow rehabilitation studies on specific outcomes, helping HCP by identifying a knowledge base that could be used to enable individualization in clinical practice, requires a more comprehensive approach to individualized rehabilitation. Stout et al. (2012) [58] stated that the current model of care often lacks attention to BC patients’ physical and functional wellbeing and have developed a model for prospective surveillance focusing on physical and functional limitations. Their model provides a broad approach to physical and functional rehabilitation that includes evaluation, education, re-assessment and ongoing surveillance for early identification and management of impairments. This is in line with the current study’s comprehensive approach to rehabilitation.

Most SRs included in this study were related to lymphoedema treatment and several treatments were investigated. Lymphoedema is a common problem following BC treatment. According to Stuiver et al. (2015) [47], preventive strategies seem to be more beneficial when started earlier rather than late. This indicates the importance of close and frequent monitoring after BC surgery. Patients with lymphoedema often require specialist care and resistance training appears to be safe and beneficial, mainly for volume reduction and increased muscle strength. However, the complexity in this area calls for team interventions with an individualized approach.

In the present review, Tai Chi was the intervention type that failed to show positive effects apart from short-term beneficial effects on upper limb functional mobility. Also, despite promising results for mindfulness-based stress reduction, acupoint stimulation and massage, more high-quality studies in these areas are needed.

Rehabilitation interventions are designed to optimize functioning and reduce disability in individuals with health conditions in interaction with their environment. Finding a way to adapt to the new life situation and return to work is therefore a complex but important question both for the individual and for society. However, evidence on effective interventions to support return to work among patients with BC is sparse and it has been stated that employment status and work performance is associated with a combination of individual factors, the work environment, culture, and resources [59], indicating the need for individualized rehabilitation. It is also well known that the rehabilitation needs following BC treatment vary greatly among individuals. Since BC is common and many women are in need of rehabilitation, individualization becomes a major issue in the effort to optimize rehabilitation and use available resources as effectively as possible. To enable this, it is fundamental to provide clinicians with extended knowledge about the effectiveness of different interventions for specific outcomes. Within the BC rehabilitation area a great amount of research is available. However, there is still a gap between rehabilitation research and practice which emphasis further research focusing on dissemination and implementation of available research findings. This SR of SRs extends the knowledge base by providing a comprehensive review of the effectiveness of these interventions.

### Strengths and limitations

One way to make evidence available to HCPs and clinical decision makers is by providing them with a summary of available evidence through a SR of SRs. The purpose of such a review is to identify and review all published reviews within a specific field and rate their quality, summarize and compare their conclusions, and discuss the strength of these [14]. Through such an approach, the present SR of SRs provides a comprehensive evidence base for the development of individualized rehabilitation. This is greatly needed in the area of BC rehabilitation, which has a large number of available studies with often divergent interventions and results.

Systematic reviews evaluating the effect of healthcare interventions often include both RCTs and NRSI. Therefore, this SR of SRs used the AMSTAR 2 tool [15] that is a critical appraisal tool for systematic reviews that were developed to evaluate methodological quality in both RCTs and NRSI. To assure interrater agreement, Kappa coefficient were evaluated and 14 out of 18 evaluated items were graded as having a perfect or substantial agreement which indicate a small difference between raters. Assessing the methodological quality is necessary to establish a sound foundation for the analysis and results. However, the AMSTAR 2 tool consists of some items that could be of limited relevance/or are not described in the included SRs. This means that SR within the rehabilitating area are likely to get a lower grading due to e.g. that blinding is only possible at allocation and outcome level or that comparators are not described since it is considered the absence of the intervention. This is likely to affect the methodological grading and needs to be taken in account when interpreting the results.

A potential limitation of this SR of SRs is that when including both SR and meta-analyses there is a risk that the same studies may have been included in more than one SR. Therefore, we chose not to draw conclusions on the number of SRs presented within each area or based on the study design. However, the inclusion of both SRs and meta-syntheses enables a broader scope and a more comprehensive approach to BC rehabilitation compared with other SRs.

Another limitation is the variation in and descriptions of the included cohort of patients within each SR, for example regarding where in the cancer trajectory the patients were, whether they were undergoing active cancer treatment or not and what kind of treatment they had received. Based on this variation, an evaluation of effects was only identified within some of the areas and was then related to where the patients were in their cancer trajectory. This is a limitation and needs to be taken into consideration when interpreting the results and it emphasizes the need for further studies in the field. However, we included the SRs to enable a broader description of rehabilitation interventions in this patient population.

The level of detail in the description of the results in the various areas is based on the heterogeneity of the included interventions. For example, yoga, compared with the other rehabilitation areas, may be considered a fairly homogeneous intervention and therefore subgroup analyses are possible, enabling a more detailed description of who needs what, and when. On the other hand, CAM interventions are heterogeneous, which prevents detailed descriptions that are of clinical relevance.

## Conclusions

The result summarizes the available evidence and underpins findings of the positive effects of exercise and PA and yoga for women following BC treatment. It also extends knowledge about the effects of CAM, lymphoedema treatment and psychosocial interventions in BC rehabilitation. It is evident that more than one intervention could have positive effects on a specific symptom or problem and that the effects depend not only on intervention type, but also on how and when the intervention is provided. The results could be used as a foundation for individualized rehabilitation and may aid HCPs in meeting patients’ individual needs and preferences.

## Additional file

Additional file 1: Search strategy. (DOCX 26 kb)

## Abbreviations

BC: Breast cancer
BMI: Body mass index
CAM: Complementary alternative medicine
CBT: Cognitive behavioral therapy
HCP: Health care professional
HRQoL: Health-related quality of life
MLD: Manual lymphatic drainage
PA: Physical activity
QoL: Quality of life
RCT: Randomized controlled trial
