# Supplementary material for: A comprehensive approach to rehabilitation interventions following breast cancer treatment - a systematic review of systematic reviews
Source: BMC Cancer. 2019 May 20;19:472. doi: 10.1186/s12885-019-5648-7 (PMC6528312; doi:10.1186/s12885-019-5648-7)
Supplement: Supplementary file 1 — Search strategy. (DOCX 26 kb) [file 12885_2019_5648_MOESM1_ESM.docx]

**Rehabilitation after breast cancer surgery – Systematic reviews and meta analyses**

Literature search in Embase, PubMed, Cinahl Complete, PsycInfo, AMED, Scopus, Cochrane Library.

Searches performed 2017-09-30. Matthias Bank, Faculty of Medicine, Lund University.

Restriction to systematic reviews and meta analyses, English language, items with abstract.

| **Search results overview** | |
| --- | --- |
| **Database** | **Number of hits** |
| AMED | 14 |
| Cinahl Complete | 102 |
| Cochrane Library   - Cochrane Systematic Reviews - Technology Assessments | 27  6 |
| Embase | 356 |
| PsycInfo | 41 |
| PubMed | 547 |
| Scopus | 176 |
| **Sum** | **1269** |
| **Number of items after deduplication** | **936** |

**AMED, 2017-09-30**

#1 ( "lymph node dissection" OR surgery OR postsurgery OR "post surgery" OR "post surgical" OR postsurg* OR "post operative" OR postoperative OR mastectomy OR "breast resection" OR lumpectomy )  15,216

#2 "Breast Neoplasms" OR "breast cancer"  1,514

#3 #1 AND #2 212

#4 breast cancer rehabilitation 285

#5 #3 OR #4 430

#6 ( (DE "Rehabilitation" OR DE "Cognitive Rehabilitation" OR DE "Occupational Therapy" OR DE "Physical Therapy" OR DE "Psychosocial Rehabilitation") OR (DE "Exercise") ) OR ( rehabil* OR physiothera* OR exercise OR aerobic* OR "physical activity" OR sport OR yoga OR mobilization OR stretching OR massage OR training OR acupuncture )  120,017

#7 ( (((DE "Psychologists") OR (DE "Social Workers")) OR (DE "Nurses")) OR (DE "Cognitive Therapy") ) OR ( psychologist* OR "social worker" OR "social workers" OR "case worker" OR "case workers" OR nursing OR nurse* OR mindfulness OR "stress management" OR "cognitive therapy" OR "cognitive behavioral" OR "cognitive behavioural" OR cbsm OR cbt OR psychosocial OR kinesiotherapy OR myofascial )  21,940

#8 “cognitive behavioral stress management” OR “patient assistance therapy” OR “group training” OR “group therapy” OR “occupational therapy” OR “occupational therapist” OR “occupational therapists”  13,622

#9 #6 OR #7 OR #8 139,746

#10 #5 AND #9 362

#11 (#5 AND #9 ) AND (TI ( "meta-analysis" OR "meta-analyses" OR "systematic review" OR "systematic reviews" OR cochrane ) OR AB("meta-analysis" OR "meta-analyses" OR "systematic review" OR "systematic reviews" OR Cochrane), 14

**Cinahl Complete, 2017-09-30**

#1 ( (MH "Lymph Node Excision+") OR (MH "Surgery, Operative+") OR (MH "Mastectomy+") OR (MH "Lumpectomy") ) OR ( "lymph node dissection" OR surgery OR postsurgery OR "post surgery" OR "post surgical" OR postsurg* OR "post operative" OR postoperative OR mastectomy OR "breast resection" OR lumpectomy )  625,616

#2 (MH "Breast Neoplasms+") OR "breast cancer"  74,008

#3 #1 AND #2 15,154

#4 breast cancer rehabilitation 843

#5 #3 OR #4 15,703

#6 ( (MH "Rehabilitation+") OR (MH "Rehabilitation Nursing") OR (MH "Physical Therapy+") OR (MH "Exercise+") ) OR ( rehabil* OR physiothera* OR exercise OR aerobic* OR "physical activity" OR sport OR yoga OR mobilization OR stretching OR massage OR training OR acupuncture )  579,562

#7 ( (MH "Psychologists") OR (MH "Social Workers") OR (MH "Nurses+") OR (MH "Cognitive Therapy+") OR (MH "Rehabilitation, Cancer") ) OR ( psychologist* OR "social worker" OR "social workers" OR "case worker" OR "case workers" OR nursing OR nurse* OR mindfulness OR "stress management" OR "cognitive therapy" OR "cognitive behavioral" OR "cognitive behavioural" OR cbsm OR cbt OR psychosocial OR kinesiotherapy OR myofascial )  1,106,779

#8 (MH "Occupational Therapy+") OR ( “cognitive behavioral stress management” OR “patient assistance therapy” OR “group training” OR “group therapy” OR “occupational therapy” OR “occupational therapist” OR “occupational therapists” )  36,732

#9 #6 OR #7 OR #8 1,576,835

#10 #5 AND #9 3,519

#11 #10, Limit English Language; Publication Type: Meta Analysis, Systematic Review 70

#12 #5 AND #9 AND (TI ( "meta-analysis" OR "meta-analyses" OR "systematic review" OR "systematic reviews" OR cochrane ) OR AB("meta-analysis" OR "meta-analyses" OR "systematic review" OR "systematic reviews" OR Cochrane), Limit English Language 80

#13 #11 OR #12 102

**Cochrane Library, 2017-09-30**

Title: "breast cancer" OR "breast neoplasms"
AND
Title, abstract, keywords: rehabilitation OR rehabil* OR physiotherapy OR exercise OR aerobic OR "motor activity" OR “physical activity” OR sport* OR yoga OR mobilization OR stretching OR massage OR training OR acupuncture OR psychologist* OR “social worker” OR “social workers” OR “case worker” OR “case workers” OR nursing psychologist* OR "social worker" OR "social workers" OR "case worker" OR "case workers" OR nursing OR nurse OR nurse* OR mindfulness OR "stress management" OR "cognitive therapy" OR "cognitive behavioral" OR "cognitive behavioural" OR cbsm OR cbt OR psychosocial OR kinesiotherapy OR myofascial OR "cognitive behavioral stress management" OR "patient assistance therapy" OR "group training" OR "group therapy" OR "occupational therapy" OR "occupational therapist" OR "occupational therapists"

Cochrane Reviews: 27
Technology Assessments: 6

**Embase strategy 2017-09-30**

#1 'lymph node dissection'/exp OR 'lymph node dissection' OR 'cancer surgery'/exp OR 'cancer surgery' OR 'surgery'/exp OR surgery OR 'post surgery' OR postsurg* OR 'post surgical' OR 'post operative' OR postoperative OR 'mastectomy'/exp OR mastectomy OR 'breast resection'/exp OR 'breast resection' OR 'lumpectomy'/exp OR 'lumpectomy' 5,723,223

#2 'breast cancer'/exp OR 'breast cancer' 460,626

#3 #1 AND #2 146,652

#4 'breast cancer' NEAR/4 rehabilitation 568

#5 #3 OR #4 146,911

#6 'rehabilitation'/exp OR rehab* OR 'physiotherapy'/exp OR physiothera* OR 'exercise'/exp OR exercise OR aerobic* OR 'physical activity'/exp OR 'physical activity' OR 'sport'/exp OR sport OR yoga OR mobilization OR stretching OR massage OR 'training' OR acupuncture 2,088,315

#7 'psychologist'/exp OR psychologist* OR 'psychological and psychiatric procedures'/exp OR 'social worker'/exp OR 'social worker' OR 'social workers' OR 'case worker' OR 'case workers' OR 'oncology nursing'/exp OR 'nursing'/exp OR nursing OR 'nurse'/exp OR nurse* OR mindfulness OR 'stress management' OR 'cognitive therapy'/exp OR 'cognitive therapy' OR 'cognitive behavioral' OR 'cognitive behavioural' OR cbsm OR cbt OR psychosocial OR kinesiotherapy OR myofascial 2,099,465

#8 'cognitive behavioral stress management'/exp OR 'patient assistance therapy' OR 'group training' OR 'group therapy' OR 'occupational therapy'/exp OR 'occupational therapy' OR 'occupational therapist' OR 'occupational therapists' 65,124

#9 #6 OR #7 OR #8 3,833,961

#10 #5 AND #9 12,687

#11 #5 AND #9 AND ([cochrane review]/lim OR [systematic review]/lim OR [meta analysis]/lim) AND [english]/lim AND [abstracts]/lim 330

#12 #5 AND #9 AND ('meta-analysis':ti,ab OR 'meta-analyses':ti,ab OR 'systematic review':ti,ab OR 'systematic reviews':ti,ab OR cochrane:ti,ab) AND [english]/lim AND [abstracts]/lim 356

#13 #11 OR #12 426

#14 #13 AND ('article'/it OR 'article in press'/it OR 'review'/it) 356

**PsycInfo, 2017-09-30**

#1 ( (DE "Surgery") OR (DE "Mastectomy") ) OR ( "lymph node dissection" OR surgery OR postsurgery OR "post surgery" OR "post surgical" OR postsurg* OR "post operative" OR postoperative OR mastectomy OR "breast resection" OR lumpectomy )  48,995

#2 DE "Breast Neoplasms" OR "breast cancer"  11,994

#3 #1 AND #2 1,905

#4 breast cancer rehabilitation 548

#5 #3 OR #4 2,330

#6 ( (DE "Rehabilitation" OR DE "Cognitive Rehabilitation" OR DE "Occupational Therapy" OR DE "Physical Therapy" OR DE "Psychosocial Rehabilitation") OR (DE "Exercise") ) OR ( rehabil* OR physiothera* OR exercise OR aerobic* OR "physical activity" OR sport OR yoga OR mobilization OR stretching OR massage OR training OR acupuncture )  591,165

#7 ( (((DE "Psychologists") OR (DE "Social Workers")) OR (DE "Nurses")) OR (DE "Cognitive Therapy") ) OR ( psychologist* OR "social worker" OR "social workers" OR "case worker" OR "case workers" OR nursing OR nurse* OR mindfulness OR "stress management" OR "cognitive therapy" OR "cognitive behavioral" OR "cognitive behavioural" OR cbsm OR cbt OR psychosocial OR kinesiotherapy OR myofascial )  500,504

#8 “cognitive behavioral stress management” OR “patient assistance therapy” OR “group training” OR “group therapy” OR “occupational therapy” OR “occupational therapist” OR “occupational therapists”  31,581

#9 #6 OR #7 OR #8 1,002,063

#10 #5 AND #9 1,271

#11 #10, Limit English, Methodology: meta analysis, systematic review 32

#12 (#5 AND #9 ) AND (TI ( "meta-analysis" OR "meta-analyses" OR "systematic review" OR "systematic reviews" OR cochrane ) OR AB("meta-analysis" OR "meta-analyses" OR "systematic review" OR "systematic reviews" OR Cochrane), Limit English Language 35

#13 #11 OR #12 41

**PubMed strategy 2017-09-30**

#1 "Lymph Node Excision"[Mesh] OR “lymph node dissection” OR surgery or “surgery”[mesh] OR postsurgery OR “post surgery” OR “post surgical” OR postsurg* OR “post operative” OR postoperative OR mastectomy OR “breast resection” OR lumpectomy 4,205,579

#2 “breast neoplasms”[Mesh] OR “breast cancer” 316,427

#3 #1 AND #2 92,769

#4 “breast cancer rehabilitation” 20

#5 #3 OR #4 92,781

#6 “rehabilitation”[mesh] OR rehabil* OR physiotherapy OR physiotherapy* OR exercise OR aerobic OR “motor activity”[mesh] OR “physical activity” OR sport OR yoga OR mobilization OR stretching OR massage OR training OR acupuncture 9,208,313

#7 psychologist OR psychologists OR “social worker” OR “social workers”[mesh] OR “case worker” OR “case workers” OR nursing OR nurse OR nurse* OR mindfulness OR “stress management” OR “cognitive therapy”[mesh] OR “cognitive therapy” OR “cognitive behavioral” OR “cognitive behavioural” OR cbsm OR cbt OR psychosocial OR kinesiotherapy OR myofascial 1,965,570

#8 “cognitive behavioral stress management” OR “patient assistance therapy” OR “group training” OR “group therapy” OR “occupational therapy”[mesh] OR “occupational therapy” OR “occupational therapist” OR “occupational therapists” 50,561

#9 #6 OR #7 OR #8 10,527,413

#10 #5 AND #9 24,487

#11 #10, Limit: Article types Meta-analysis, Systematic reviews, English, has abstract 509

#12 #10 AND ("meta-analysis"[Title/Abstract] OR "meta-analyses"[Title/Abstract] OR "systematic review"[Title/Abstract] OR "systematic reviews"[Title/Abstract] OR cochrane[Title/Abstract]), Filters English, has abstract 330

#13 #11 OR #12 547

**Scopus, 2017-09-30**

#1 ( TITLE-ABS-KEY ( "Lymph Node Excision"  OR  "lymph node dissection"  OR  surgery  OR  postsurgery  OR  "post surgery"  OR  "post surgical"  OR  postsurg*  OR  "post operative"  OR  postoperative  OR  mastectomy  OR  "breast resection"  OR  lumpectomy )  AND  TITLE-ABS-KEY ( "breast neoplasms"  OR  "breast cancer" ) )  77,376

#2 TITLE-ABS-KEY ( "breast cancer"  W/4  rehabilitation )  232

#3 #1 OR #2 77,479

#4 ( TITLE-ABS-KEY ( "rehabilitation"  OR  rehabil*  OR  physiotherapy*  OR  exercise  OR  aerobic  OR  "physical activity"  OR  sport  OR  yoga  OR  mobilization  OR  stretching  OR  massage  OR  training  OR  acupunctur* ) ) 2,162,303

#5 ( TITLE-ABS-KEY ( psychologist*  OR  "social worker"  OR  "social workers"  OR  "case worker"  OR  "case workers"  OR  nursing  OR  nurse*  OR  mindfulness  OR  "stress management"  OR  "cognitive therapy" )  OR  TITLE-ABS-KEY ( "cognitive behavioral"  OR  "cognitive behavioural"  OR  cbsm  OR  cbt  OR  psychosocial  OR  kinesiotherapy  OR  myofascial ) ) 1,065,798

#6 ( TITLE-ABS-KEY ( "cognitive behavioral stress management"  OR  "patient assistance therapy"  OR  "group training"  OR  "group therapy"  OR  "occupational therapy" )  OR  TITLE-ABS-KEY ( "occupational therapist"  OR  "occupational therapists" ) )  57,771

#7 #4 OR #5 OR #6 3,099,488

#8 #3 AND #7 4,787

## #9 TITLE-ABS-KEY ( "meta-analysis"  OR  "meta-analyses"  OR  "systematic review"  OR  "systematic reviews"  OR  cochrane )   313,685

#10 #8 AND #9 193

Limit: English, publication types, articles, articles in press, reviews 176
